# Poor sleep quality and associated factors among prisoners of the Diredawa correctional facility in eastern Ethiopia

**DOI:** 10.1186/s12991-020-00291-6

**Published:** 2020-06-20

**Authors:** Yibeltal Getachew, Telake Azale, Mogesie Necho

**Affiliations:** 1 Department of Psychiatry, College of Medicine and Health Sciences, Diredawa University, Diredawa, Ethiopia; 2 Department of Psychiatry, College of Medicine and Health Sciences, University of Gondar, Gondar, Ethiopia; 3 Department of Psychiatry, College of Medicine and Health Sciences, Wolli University, Dassie, Ethiopia

**Keywords:** Quality of sleep, Prisoner, Correctional facility, Diredawa

## Abstract

**Background:**

Impaired sleep quality affects judgment, psychomotor skills, memory, decision-making, concentration, and attention. It might also contribute to the development of new physical health problems, as well as exacerbating already existing physical problems. Despite this, there is a scarcity of research done in Africa including Ethiopia that addressed this issue. So this study assessed the quality of sleep and related factors among prisoners of the Diredawa correctional facility, Diredawa, eastern Ethiopia.

**Methods:**

A cross-sectional study was done using a simple random sampling technique to recruit 421 participants from May 21 to June 21; 2017. A semi-structured questionnaire, Pittsburgh Sleeps Quality Index (PSFIG), Patient Health Questionnaire-9 (PQ-9), and Sleep Hygiene Index (SHI) were used to assess participants’ socio-demographic data, sleep quality, depression, and sleep hygiene, respectively. The collected data were entered into EPA-data 3.1 and analyzed by using SPAS-20. Descriptive and analytical statistics were used. Bivariate and multivariable logistic regression with odds ratio and 95% CI were employed. The statistical significance was declared at *p* value < 0.05.

**Results:**

A total of 421 out of 423 prisoners were interviewed, resulting in a response rate of 99.5%. The prevalence of poor sleep quality was 227 (53.9%) with 95% CI (49.2, 58.7). Having co-morbidity of depression (adjusted odds ratio; OAR = 3.47, 95% CI 1.38,8.76), lifetime use of cigarette (OAR = 2.16, 95% CI 1.21,5.58), marijuana and hashish (OAR = 5.02, 95% CI 1.63,15.46), current use of coffee (OAR = 2.75, CI 1.37, 7.05), poor sleep hygiene (OAR = 3.19, CI 1.32,7.69), committing assault crime (OAR = 4.12, CI 1.29,10.63) and crime of rape (OAR = 5.57, CI (1.45, 13.89) were the associated factors for poor sleep quality in this study.

**Conclusion:**

More than half of the participants (53.9%) have poor sleep quality. Depression, lifetime use of cigarettes, using cannabis and hashish, current use of coffee, poor sleep hygiene, and crime types were the associated factors that should be taken into consideration and evaluated early to minimize poor sleep quality.

## Background

Sleep occupies one-third of our lives and is essential to physical and mental growth and stability. Its deprivation weakens physical and mental functions, lowers work productivity, and can cause mental problems such as depression [1]. The duration, quality, and timing of sleep are essential elements to have adequate sleep. Partial sleep loss is common in many segments of the population, having enormous effects both on society and individual levels [1, 2].

Sleep deprivation has serious health consequences including problems related to performance, daytime sleepiness, and fatigue as short-term and long-term effects related to premature mortality, cardiovascular disease, hypertension, inflammation, obesity, diabetes and impaired glucose tolerance, and psychiatric disorders, such as anxiety and depression. Moreover, it affects mental processes and intellectual abilities, impedes decision-making and memory, and reduces performance on challenging tasks and has negative effects on psychomotor skills, mood, productivity, and communication skills. Chronic lack of sleep can contribute to serious health problems and a shortened lifespan [1, 3–6].

Sleep disturbances are prevalent in prisoners than in the general population, 30%-36% higher [7, 8]. A study in Tartu, Estonia, discovered poor sleep quality in 62% of elderly prisoners [9]. Similarly, a study in the US highlighted that poor sleep quality ranged from 52 to 60% in male prisoners [10]. Furthermore, another US study revealed poor sleep quality in 62.7% of men and 81.2% of women [11]. Moreover, poor sleep quality among prisoners was 88.2% in UK [12], 44% in a Swiss jail [13], 87.5% in Switzerland [14], 61.2% in Italy [15], 41.2% in Norway [16], 73% in South Africa [17] and 37.7% in Nigeria [18].

A study of prisoners in Switzerland reported that prisoners with a low level of education, who were unemployed, separated, or divorced had higher rates of poor sleep quality [19, 20]. In addition, behavioral factors like poor sleep hygiene, watching television at night for a long period, taking caffeinated drinks and napping during the day are the factors related to poor sleep quality of prisoners [18, 20]. Moreover, anxiety related to the situation, fear of violence, forced contact with others and being worried [7, 12, 19–23], night-shift work [21], depressive disorders [24, 25], prolongation of detention or long-term offenders [26, 27], substance use [18, 20], committing a serious crime [28], and lack of physical activity [18] were the related factors for poor sleep quality in prisoners.

Poor sleep quality in prisoners aggravates an already existing medical problem, leads to the rise of new health problems, and weakens a detainee’s ability to fruitfully contribute [23]. It has also an impact on cognitive performance, psychosocial functioning, and the perception of stress [22, 29]. Moreover, prisoners with poor sleep quality have a risk of aggressive and violent behavior [30–32], suicidal behaviors [15, 33, 34], depression [35, 36], and vascular complications [37, 38] in diabetic prisoners.

However, studies in the sleep quality of prisoners in correctional institutions of Africa are limited and as per the knowledge of investigators, no research has been done in Ethiopia. Such a shortage of literature and evidence challenges the management of sleep problems in prison settings and health care professionals have a variety of very different approaches to deal with these complaints and most of the time underestimate the problem and consider it as normal.

This study, therefore, assessed the prevalence and related factors of poor sleep quality of prisoners in the Diredawa correctional facility. Besides, the evidence obtained from this study will serve as a baseline for further studies in the area. The objectives of this study were, therefore, to determine the prevalence of poor sleep quality and identify its associated factors among prisoners. Our reason to study this public health issue is that the Ethiopian Federal Ministry of Health considered prisoners as vulnerable groups for mental health disorders, including sleep problems.

## Methods

### Study setting and period

This institutional cross-sectional study was implemented from May 21 to June 21, 2017, at Diredawa correctional facility. Diredawa correctional facility is located in Diredawa city administration, 515 km away from Addis Ababa. It is one of the five federal correctional facilities that receive prisoners who are both sentenced and awaiting the court’s decision. The institution has a capacity of holding about 3000 prisoners and at the time of the data collection, there were 1500 prisoners, of which 1356 have gotten the court’s decision and the rest 144 were awaiting a trial. Of the sentenced prisoners, 1265 were male and 91 were female. The prison receives nearly all men and women prisoners. The prison population in common with other federal prisons is unstable with large numbers of prisoners being received and discharged almost every day.

### Study participants

The sample size for this study was estimated using the single population proportion formula. The assumptions taken into consideration during calculation of sample size include: (1) prevalence of poor sleep quality to be 50% since no earlier study in Ethiopia; (2) the *Zα*⁄2-value of 1.96 at a 95% confidence interval; (3) a margin of error of 5%, and (4) a non-response rate of 10%. Finally, a total of 423 prisoners were planned to be included in the study. Since the total number of male and female sentenced prisoners was 1265 and 91, we considered proportional allocation to male and female prisoners and 393 male and 28 female prisoners were allowed to participate. A simple random sampling technique was used to select study units.

Our source population was all the prisoners found in Diredawa federal prison, whereas the study population comprised all sentenced prisoners in Diredawa federal prison who had got the chance to be eligible for inclusion of data collection during the study period. We invited all prisoners who have got a court decision to be included in the study, but prisoners who had an already known diagnosed psychiatric problem such as bipolar disorder and anti-social as well as borderline personality disorders were excluded since this might overestimate the magnitude of poor sleep quality. Also, prisoners who were in the isolation room and having chronic physical illness were not allowed to participate in the study since such conditions might also affect the quality of sleep and overemphasize its magnitude (see Additional file 1).

### Operational definitions

Sleep quality: using the Pittsburgh sleep quality index (PSFIG) at cut-off point greater than 5, we screened poor sleep quality, whereas good sleep quality was a score less than or equal to 5 in this scale [39].

Depression: according to PQ-9, prisoners scoring ≥ 5 were screened as having depression [40].

Anxiety: a score of ≥ 8 on the GAD-7 scale was considered as a general anxiety disorder [41].

Poor sleep hygiene: a score above the mean on sleep hygiene index scale was categorized as poor sleep hygiene; the mean score in this study was 14 [42].

Lifetime use of substances: use of substances such as tobacco, khat, alcohol, and other substances (marijuana and hashish) for non-medical purposes before they become imprisoned.

Current use of caffeinated drinks: use of caffeinated drinks for non-medical purpose within the previous 3 months.

### Instruments used for data collection and data collection procedures

Sleep quality was assessed by using the Pittsburgh Sleep Quality Index (PSFIG), a self-report instrument comprising 19 items evaluating seven components of sleep over the past month: subjective sleep quality, sleep latency, sleep duration, habitual sleep efficiency, sleep disturbances, daytime dysfunction and use of sleep medications. Each component is scored (range 0–3; higher scores indicating worse sleep).

A total global PSFIG score ranges from 0 to 21, higher scores indicating poor sleep quality and A global PSFIG score > 5 was used as cut-off point with a diagnostic sensitivity of 89.6% and specificity of 86.5% (*κ* = 0.75, *p* ≤ 0.001) in distinguishing “good” from “poor” sleepers [39]. The Pittsburgh Sleep Quality Index was chosen since it can assess the past one-month prevalence of sleep problems and the information obtained would be essential to initiate intervention measures for the prisoners with sleep disturbance.

Patient Health Questionnaire (PQ-9) developed by Drs. Robert L. Spitzer, Janet B.W. Williams, Kurt Koneke, and colleagues was used to screen depression in prisoners in the last 2 weeks. The PQ-9 severity score ranges from 0 to 27, since the 9 elements ranged from 0 (“not at all”) to 3 (“nearly every day”). The cut-off points of 5, 10, 15, and 20 represent the thresholds for mild, moderate, moderately severe, and severe depression, respectively [40].

The Sleep Hygiene Index (SHI) is a 13-item self-report measure designed to assess the practice of sleep hygiene behaviors. Each item is rated on a five-point scale ranging from 0 (never) to 4 (always). An overall score varies from 0 to 52, with a greater score demonstrating reduced sleep hygiene. SHI has revealed acceptable reliability and validity. The SHI has revealed modest internal consistency [42]. Data regarding criminal offenses of each prisoner were obtained from a retrospective recall of the prisoners.

Data quality was ensured by training data collectors, closely following the data collection process, and checking the completeness of filled questionnaire at the end. Besides, the questionnaire was pretested on 5% [18] of the detainees in a jail found in Diredawa city administration. Based on the finding of the pretest, there were words in the data collection questionnaire which were wrongly perceived by the participants (e.g., when they were asked what was their religion, some chose “Amhara” to represent the Orthodox Christian religion. Therefore, all data collectors discussed and considered this during the main data collection period.

### Data processing and analysis

The collected data were entered into DATA 3.1 software and then exported to Statistical Package for Social Sciences version 20 (SPAS-20) software for analysis. Descriptive statistics (frequencies, percentages, cross-tabulations) were used to describe sleep quality and its associated factors. Bivariate and multivariable logistic regression analyses were conducted to identify associated factors of sleep quality. All independent variables with *p* value < 0.25 on bivariate analysis were fitted into a multivariable logistic regression to control the confounder’s effect. The level of significance at the final model was declared at a *p*-value of < 0.05 and a 95% confidence interval.

### Ethical consideration

Ethical clearance was obtained from the ethical institutional review board (EIRB) of the University of Gondar (UOG) and an ethical review committee of Amanuel Mental Specialized Hospital (AMSH) and submitted to Diredawa federal prison administration. The purpose and importance of the study were explained to each participant before they proceed into actual activities. While informing prisoners about the study, particular attention was placed in clarifying that no advantages or disadvantages for the subject would come from accepting or refusing to participate in the study. Finally, they were asked about their willingness to participate and written consent was obtained. One participant who was depressed and suicidal and having a poor sleep quality with psychological distress was linked to the Dill Chora referral hospital psychiatry unit.

## Results

### Socio-demographic characteristics of respondents

Of the 423 invited prisoners, 421 had been interviewed resulting in a response rate of 99.5%. The majority, 393 (93.3%) of the respondents, were male. The mean age of the respondents was 31.35 years with SD (± 10.33) and a range of 18 to 72 years. Three-fourths of the surveyed prisoners, 312 (74.1%) were in the age groups of 20–39 years. Of the participants, 192 (45.6%) were Muslims and 167 (39.7%) of the participants were Oromo. The majority, 179 (42.6%) of the participants were single and most of the participants 164 (39%), attended primary school as shown below (Table 1).

**Table 1:** Socio-demographic characteristics of prisoners in Diredawa correctional facility, Diredawa, Eastern Ethiopia, 2017 (*n* = 421)

Independent variable	Category	Frequency	Percent
Age in years	≤ 19	28	6.7
	20–39	312	74.1
	40–59	70	16.6
	≥ 60	11	2.6
Sex	Male	393	93.3
	Female	28	6.7
Ethnicity	Amhara	136	32.3
	Oromo	167	39.7
	Others^a^	118	28
Marital status	Single	179	42.5
	Married	176	41.8
	Others^b^	66	15.7
Religion	Orthodox	170	40.4
	Muslim	192	45.6
	Others^c^	59	14
Educational status	Can’t read and write	48	11.4
	Primary	164	39
	Secondary	147	34.9
	Diploma and above	62	14.7
Average monthly income of family	< $27	159	37.8
	$27–$43.56	96	22.8
	> $43.56	166	39.4
Social support	Poor	128	30.4
	Moderate	230	54.6
	Good	63	15

^a^Tigre, Somali, Afar, Garage. ^b^Divorced, widowed. ^c^Protestant, catholic, wakifeta

### Psychological and behavioral variables of prisoners in Diredawa correctional facility

Among the interviewed prisoners, 266 (63.2%) were obtained to have depression and 149 (35.4%) had general anxiety disorder symptoms. The lifetime use of substances among the study participants was 306 (72.7%). Nearly two-thirds of participants, 271 (64.4%), used Khat in their lifetime. Also, approximately three-fifths, 260 (61.8%), of participants are currently using coffee. Regarding the sleep hygiene of participants, 263 (62.5%) participants had poor sleep hygiene. Considering sex, 14 (50%) of female participants and 249 (63.36%) of male participants had poor sleep hygiene (Table 2).

**Table 2:** Psychological and behavioral factors of prisoners in Diredawa correctional facility, Diredawa, Eastern Ethiopia, 2017

Independent variables	Category	Frequency (*n*)	Percent (%)
Depression	Yes	266	63.2
	No	155	36.8
Life time substance use	Yes	306	72.7
	No	115	27.3
Alcohol	Yes	224	53.2
	No	197	46.8
khat	Yes	271	64.4
	No	150	35.6
Cigarette smoking	Yes	194	46.1
	No	227	53.9
Other substances^a^	Yes	51	12.1
	No	370	87.90
Caffeinated drinks	Yes	315	74.8
	No	106	25.2
Spris	Yes	61	14.5
	No	360	85.5
Coffee	Yes	260	61.8
	No	161	38.2
Coca cola	Yes	171	40.6
	No	250	59.4
Pepsi	Yes	23	5.5
	No	398	94.5
Poor sleep hygiene	Yes	263	63.5
	No	158	36.5

^a^Other substances include marijuana, hashish

### Criminal and work-related variables of sleep quality among prisoners

The frequency of almost all crime types was close to each other which were theft (15.2%), murder/attempted murder (15.2%), fraud (17.3%), assault (15.9%), rape (14.3%) and other crimes (22.1%). Among the prisoners 5.5% remained in prison for more than 18 years and the majority, 267 (63.4%), were in the prison between 1 and 9 years. Of the participating prisoners, 144 (34.2%) had work shifts including both night and day shifts and 277 (65.8%) did not participate in any income-generating activities within the prison (Table 3).

**Table 3:** Criminal and work-related factors of prisoners in Diredawa correctional facility, Diredawa, Eastern Ethiopia, 2017 (*n* = 421)

Independent variable	Category	Frequency (*n*)	Percent (%)
Crime type	Violent crime	131	45.4
	Non-violent crime	137	32.5
	Others^a^	93	22.1
Duration of sentence	≤ 1 year	48	11.4
	1–5 years	162	38.46
	6–10 years	105	24.94
	11–18 years	83	19.7
	≥ 18 years	23	5.5
Day work shift	Yes	134	31.8
	No	287	68.2
Night work shift	Yes	10	2.4
	No	411	97.6

Violent crime: murder, assault and rape

Non-violent crime: theft and fraud

^a^Others—burglary, terrorism, illegal immigration, traffic accident, contraband, guy, cheating, not following principle, not respecting appointment, hiding people, warning people, destruction of property

### Prevalence of poor sleep quality among prisoners in Diredawa correctional institution

**Fig. 1:**
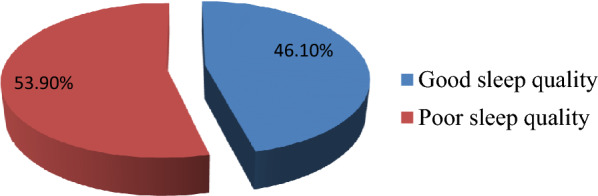
Prevalence of poor sleep quality among prisoners of Diredawa correctional facility

This study revealed that the prevalence of poor sleep quality among prisoners of the Diredawa correctional facility was 227 (53.9%) (95% CI 49.2, 58.7) (Fig. 1).

### Factors associated with poor sleep quality among prisoners in Diredawa correctional institution

In the bivariate analysis socio-demographic variables (age, sex, marital status, and educational status), behavioral factors (lifetime use of cigarette, lifetime use of khat, lifetime use marijuana or hashish, current use of caffeinated drinks, poor sleep hygiene), depression, criminal-related factors (duration of the sentence and crime type) and work-related factors (work shift) have *p* < 0.25 and then taken for further analysis into multivariate analysis.

However, only depression, lifetime use of cigarette and other substances (marijuana, and ganja or hashish), current use of coffee close to bedtime, sleep hygiene and crime types (assault and rape) were found to have a statistically significant association with poor sleep quality (*p* value < 0.05). In both the bivariate and multivariable binary logistic regression analyses, the variable group expected to be protected against poor sleep quality was treated as the reference group.

Depression was associated with poor sleep quality in this study. Prisoners who have depressive symptoms were 3.47-fold times higher to have poor sleep quality than prisoners who have no depression (OAR = 3.47, 95% CI (1.38, 8.76). Besides, the prevalence of poor sleep quality was 5.6 times higher in prisoners accused of rape (OAR = 5.57, 95% CI 1.45, 13.89) and 4 times higher in those accused of assault (OAR = 4.12, 95% CI 1.29, 10.63) as compared to other crimes.

Moreover, poor sleep hygiene practice was associated with poor sleep quality. Those participants who have poor sleep hygiene were more than three times more likely to have poor sleep quality as compared to prisoners having good sleep hygiene (OAR = 3.19, 95% CI 1.32,7.69). Concerning the respondent’s cigarette use, those who smoked cigarettes in a lifetime were more than two times more likely to develop poor sleep quality as compared with non-smokers (OAR = 2.16, 95% CI 1.21, 5.58) and those prisoners taking other substances (marijuana and hashish) had five times higher risk to have poor sleep quality as compared to those who did not use these substances in their lifetime (OAR = 5.02, 95% CI 1.63, 15.46).

Prisoners who were currently using a caffeinated drink (coffee) were almost three times more likely to have poor sleep quality as compared to non-coffee users (OAR = 2.75, 95% CI; (1.37, 7.05) (Table 4).

**Table 4:** Factors associated with poor sleep quality among prisoners of Diredawa correctional facility, Diredawa, Eastern Ethiopia, 2017 (*n* = 421)

Variables	Categories	Sleep quality		COR (95% CI)	OAR (95% CI)
		Poor (*n*/ %)	Good (*n*/ %)		
Age in years	≤ 19	8 (28.6)	20 (71.4)	1	1
	20–39	175 (56)	137 (44)	3.19 (1.36,7.47)	1.27 (0.18,9.11)
	40–59	37 (52.8)	33 (47.2)	2.80 (1.09,7.21)	0.51 (0.05,4.82)
	≥ 60	7 (63.6)	4 (36.4)	4.37 (0.99,19.16)	0.51 (0.04,7.31)
Education	Illiterate	26 (54.2)	22 (45.8)	1	1
	Grade 1–8	91 (55.5)	73 (44.5)	1.53 (0.73,3.27)	1.31 (0.38,4.50)
	Grade 9–12	83 (56.5)	64 (43.5)	1.62 (0.89,2.91)	1.03 (0.29,3.56)
	Diploma and above	27 (43.5)	35 (56.5)	1.68 (0.92,3.06)	0.61 (0.13,2.85)
Depression	No	47 (30.3)	108 (69.7)	1	1
	Yes	180 (67.7)	86 (32.3)	4.81 (3.13,7.38)	3.47 (1.38,8.76)**
Cigarette smoking	No	91 (40)	136 (60)	1	1
	Yes	129 (66.5)	65 (33.5)	2.97 (1.43,8.19)	2.16 (1.21,5.58)*
Other substance	No	111 (30)	259 (70)	1	1
	Yes	39 (76.5)	12 (23.5)	7.6 (1.21, 17.85)	5.02 (1.63,15.46)**
Coffee	No	45 (27.9)	116 (70.1)	1	1
	Yes	156 (60)	104 (40)	3.87 (1.68,10.93)	2.75 (1.37,7.05)*
Sleep hygiene	Good	51 (32.3)	107 (67.7)	1	1
	Poor	176 (66.9)	87 (30.1)	4.24 (2.79,6.47)	3.19 (1.32,7.69)**
Crime type	Theft	24 (37.5)	40 (62.5)	1	1
	Murder	30 (46.9)	34 (53.1)	1.47 (0.73,2.98)	0.77 (0.19,3.08)
	Fraud	41 (56.2)	32 (43.8)	2.14 (1.08,4.24)	2.15 (0.66,7.06)
	Assault	37 (55.2)	30 (44.8)	2.06 (1.52,4.13)	4.12 (1.29,10.63)*
	Rape	43 (71.7)	17 (28.3)	4.22 (1.98,8.98)	5.57 (1.45, 13.89)*
	Others	52 (55.9)	41 (44.1)	2.11 (1.10,4.05)	1.39 (0.46,4.19)

Key: 1.00 in the table indicates the references group during binary logistic regression analysis, *p value less than 0.05, **p value less than 0.01, Hosmer–Lemeshow (*p* = 0.80), COR: crude odds ratio, OAR: adjusted odds ratio

## Discussion

The limited available evidence regarding sleep quality in the prison settings challenges the management of prisoners with the problem. Consequently, health care professionals have a variety of approaches to deal with sleep quality problems of prisoners. This study, therefore, assessed the magnitude and related factors of poor quality of sleep among prisoners that were assumed to fill the gap in the existing evidence.

The current study found that the prevalence of poor sleep quality in prisoners was 53.9%; 95% CI (49.2, 58.7). Depression, lifetime cigarette use, use of marijuana and hashish, current use of coffee, poor sleep hygiene, and crime types (rape and assault) were the associated factors for poor sleep quality in prisoners.

The prevalence of poor sleep quality in the present study is higher than the result of a study at a Swiss jail by Elger et al. [13] in which 44% of prisoners had poor quality sleep. The current finding was also lower than the result of a study in Nigeria; 37.7% [18]. The possible reason for this variation may be due to the difference in the study population; prisoners in primary care consultations for Swiss study and male prisoners for Nigerian study, but both male and female prisoners for the current study. Besides different prison settings may have a role for the difference. Moreover, the sample size used was bigger in the Swiss study.

On the other hand, the current findings were lower than a result of a cross-sectional study in the UK [43] where 81% of female remand and 62% female sentenced prisoners had poor sleep quality. Moreover, the present finding was lower than the reported prevalence of poor sleep quality in a study from the US [10, 11], UK [7, 43], Italy [15] and South Africa [17]. This discrepancy may be due to the difference in the tool used, different administrative, and handling procedures of prisoners in different countries. Besides, constitutional differences and study design variation might play a role in such a difference.

However, the finding of the present study was consistent with a study in England where 52% of participants were reported to have had poor sleep quality [10].

Depression was found to be significantly associated with poor sleep quality in this study. Those prisoners having depression were more than 3.5 times at higher risk of having poor sleep quality as compared to non-depressed prisoners. This was supported by studies in India [8], USA [24], and Canada [27]. The possible justification could be the decrement of serotonin neurotransmitters in depressed prisoners [44, 45] that has an impact on diminished cognitive performance and quality of sleep.

Besides, lifetime substance use of cigarettes, marijuana, and hashish was significantly related to poor sleep quality. Prisoners who had a lifetime history as users of cigarettes and other substances (marijuana and hashish) were 2 and 5 times higher to have a poor quality of sleep as compared to prisoners who have no history of cigarettes and other substances used in a lifetime, respectively. This finding is supported by a study in German prison [20] and a study among the general population in Ethiopia [46]. Another study among college students in Ethiopia also obtained that caffeine consumption, cigarette smoking, and khat use are associated factors for poor sleep quality which is in line with our finding [47]. The insomniac effect of cigarettes and other substances either during use or withdrawal might be responsible for this poor quality of sleep [48].

Moreover, prisoners with current caffeine consumption were almost three times significantly higher to have a poor quality of sleep as compared to those prisoners who did not use coffee, and this is strengthened by a Nigerian study [18]. Adenosine agonists increase sedation, whereas antagonists increase insomnia [49]. Caffeine is an adenosine antagonist leading to the release of dopamine and activation of the CNS finally increasing arousal [49, 50]. Moreover, the craving or withdrawal effect of this substance in a restricted prison setting may also be responsible for the poor quality of sleep.

Participants who had poor sleep hygiene practice were more than three times more likely to have poor sleep hygiene as comparatively seen to those with good sleep hygiene consistent with a study done in Nigeria [18]. The elevated risk for poor sleep quality in participants having poor sleep hygiene could be explained by poor knowledge regarding sleep hygiene leading to poor sleep hygiene practice [51, 52] and poor sleep quality. Also, the sharing of risk factors by both poor sleep hygiene and poor quality of sleep could be responsible for this.

Moreover, the crime type was associated with poor sleep quality in the current study. Prisoners imprisoned for assault and rape were 4 and 5.6 times significantly higher to have a poor quality of sleep, respectively, as compared to prisoners accused of theft. The feelings of guilt may trouble their minds and disturb their sleep quality [28]. However, this was against a study in Finland in which committing serious crimes like murder were linked with poor quality sleep than other crimes [28]. This discrepancy may be due to the differences in the country’s constitutional laws and the handling of prisoners in prison stay.

As the association of the above-mentioned factors and poor sleep quality in this study was a snapshot presentation, further research has to be conducted to see if such hypothesis can be experimentally proven to be correct; for example, reducing the use of caffeine or the time of the use of caffeine, eliminating the use of drugs like ganja or other derivatives of cannabis, shutting off the television at a certain time, promoting sleep hygiene, creating a sense of security for each prisoner should be proven to improve the quality of sleep.

Although no association was obtained in our study, prolongation of detention was associated with sleep disturbance in a study conducted in Geneva, Switzerland [26], and Canada [27]. Such discrepancies might be due to the socio-economic and prison-related characteristics between Ethiopia and the above-indicated countries.

### Limitations of the study

This study has several limitations, some of which are outlined below. The first is the cross-sectional design of the study. Data regarding convictions and substance use of the study participants were collected retrospectively, increasing the risk of recall bias and memory lapse of the study. Moreover, the use of an assessment tool that relied on subjective measures of sleep quality was one of the limitations of this study. Besides, participants with serious mental and physical illness and those who were in isolation room who might experience more sleep problems than those who participated in this study were excluded and might underestimate the prevalence of poor sleep quality. Moreover, the lack of prior studies on prisoners’ sleep quality in the Ethiopian context makes it difficult to compare it with previous studies.

## Conclusion

More than half (53.9%) of the prisoners were FOUND to have poor SLEEP quality which implied that it is a significant public health issue that requires timely treatment. Depression, lifetime use of cigarettes, marijuana, and hashish, current use of coffee, and having poor sleep hygiene were the factors with a significant association with poor sleep quality. Therefore, improving the lifestyle of prisoners, their habits, and reducing their stressors, trauma, and exposure to physical and sexual violence should be a priority. Also, when prisoners feel the risk of abuse, they might develop a higher level of vigilance and this could lead to poor sleep quality. Improving sleep quality of prisoners might also prevent depression and substance abuse.

## Supplementary information

**Additional file 1.** A balance table for the study participant’s characteristics for inclusion and exclusion of prisoners in to the study.

## Data Availability

The data elements incorporated during this study process are available from the corresponding authors on rational requests.

## Abbreviations

AMSH: Amanuel Mental Specialized Hospital
DC: Data collector
DSM V: Diagnostic and Statistical Manual fifth edition
PHQ-9: Patient Health Questionnaire -9
PI: Principal investigator
PSQI: Pittsburgh Sleep Quality Index
QoS: Quality of Sleep
SHI: Sleep Hygiene Index
SPSS: Statistical Package for Social Sciences
UOG: University of Gondar
