# Supplementary material for: Poor sleep quality and associated factors among prisoners of the Diredawa correctional facility in eastern Ethiopia
Source: Ann Gen Psychiatry. 2020 Jun 20;19:40. doi: 10.1186/s12991-020-00291-6 (PMC7306144; doi:10.1186/s12991-020-00291-6)
Supplement: Supplementary file 1 — Additional file 1. A balance table for the study participant’s characteristics for inclusion and exclusion of prisoners in to the study. [file 12991_2020_291_MOESM1_ESM.docx]

| No | Inclusion criteria | Exclusion criteria | Remark |
| --- | --- | --- | --- |
| 1 | Age should be at least 18 years | Age less than 18 years old |  |
| 2 | Prisoners with a court decision | Prisoners awaiting a trial |  |
| 3 | Prisoners who have no earlier known psychiatric problem | Prisoners with a diagnosed known mental disorder |  |
| 4 |  | Prisoners with a diagnosed known personality disorder |  |
| 5 |  | Prisoners with a diagnosed known substance use disorder |  |
| 6 |  | Prisoners with critical illness during data collection that makes communication difficult |  |
| 7 |  | Prisoners who were in the isolation room |  |
| 8 |  | Prisoners who have chronic physical illness |  |

**Additional file 1:** A balance table for the study participant’s characteristics for inclusion and exclusion of prisoners in to the study
